# Notes and News

**Published:** 1902-02-08

**Authors:** 


### NOTES AND NEWS.

### HOSPITAL MEETINGS.

St. Mark's Hospital for Fistula.—Annual Meeting at the hospital, Thursday, February 13th, at 12 noon.

London Fever Hospital.—Annual Meeting at the hospital, Friday, February 14th, at 5.15.

Half of the Brompton Hospital for Consumption is closed for repairs, and the entertainment hall is being utilised as a ward for patients.

We are informed that the Metropolitan Hospital Saturday Fund have endowed two beds in the Eversfield Hospital, St. Leonards-on-Sea, for one year, for the use of those applying to them who are suffering from consumption.

Under the auspices of the Charity Organisation, Society, three lectures, "The Town Child," by Mr. Reginald Bray, and three lectures on "The Housing Problem," by Mr. F. W. Lawrence, will be given at the United Service Institution, Whitehall, at 4.30 p.m. on Tuesdays, from February 11th to March 18th inclusive. Information will be given by Mrs. G. Hill, 10 Kensington Mansions, Earl's Court.

On the occasion of presenting the prizes to the successful students on probation at the Army Medical School, Netley, last week, a brief address was given by Surg.-Gen. Taylor, C.B., Director-General Army Medical Service, in which he congratulated the successful students on their choice of a career. He had sometimes heard it said that both the Indian Medical Service and the Army Medical Service were not considered careers fit for men anxious to get on in their profession, but that, he said, was absolute nonsense, for in the smallest station they could go to in India they might obtain more surgical experience and practice than anywhere else. Referring to the rumour that the death-warrant of the Netley School had been signed, he could only assure his hearers that at headquarters they were entirely without information as to any proposed change, but should it ultimately be decided to remove the school to another place, he hoped that the change would work out to the benefit of the service. There were 25 successful candidates for the Indian Medical Service and *one* for the Royal Army Medical Corps!

The Glasgow Royal Infirmary is to be entirely reconstructed at an estimated cost of £170,000. That the work of the institution may be efficiently carried on in the meanwhile two temporary operating theatres and an electricity pavilion are to be added to the existing buildings.

Canada is not following the lead of the Old Country in allowing the claims of conscientious objectors. The Province of Quebec has just instituted regulations whereby all persons must be prepared to show a certificate of having been vaccinated within seven years or submit to a penalty of five dollars. Provision is made for free vaccination, so that all that can be is being done to render epidemics impossible.

At a time when foreigners on the continent, through want of knowledge, we must hope, are speaking evil of our soldiers, it is pleasant to learn that the invalid soldiers at Wynberg Hospital were entertained at Christmas by an Italian, Mr. Balesta, of the firm of Cosenza and Co. Mr. Balesta has had, as a resident in London and a visitor to South Africa, ample opportunity to know the truth, and he chose a happy way of expressing his sympathy at Wynberg.

The finances of the Radcliffe Infirmary, Oxford, are still far from being on a sound basis. Although the proceeds of a legacy were applied to meeting ordinary expenditure, a deficiency of income amounting to £458 existed at the end of last year. At the annual meeting the treasurer made a good case for the method of expenditure, stating that with due regard to efficiency all possible economy was studied. Therefore it is apparent that Oxford and its vicinity are taking from their hospital more than their due. There can be no doubt that more sources of revenue must be exploited, and that those who already give should be asked to give more, but we deprecate the suggestion that young ladies should be enlisted to beg in the streets on behalf of the charity. If the infirmary is even now not as popular as it should be, this action would make it absolutely obnoxious to many. The Metropolitan Hospital Saturday Fund has increased its revenue ever since it abolished this objectionable method of securing contributions.

A special committee has been occupied for some time in revising the rules of the Worcester Infirmary. These came up for consideration at the last meeting of the hospital committee. The rules drawn up affecting representation of collective subscribers in the management of the institution show an advanced and liberal spirit. Any society or body of persons contributing five guineas may be represented by one governor elected from their number, and in order to closely associate the working-classes with the hospital, any firm contributing £52 10s. during one year may be represented on the executive committee for one year. Representation of the working-classes on hospital boards is pushed in a number of places. Folkestone has gone beyond Worcester altogether, as the Victoria Hospital has pledged itself to try and secure the election of two members of friendly societies upon the committee of management. The friendly societies contribute only £2 2s. per annum to the hospital.

